# Trends in Invasive Infection with Methicillin-Resistant *Staphylococcus aureus,* Connecticut, USA, 2001–2010

**DOI:** 10.3201/eid1806.120182

**Published:** 2012-06

**Authors:** James L. Hadler, Susan Petit, Mona Mandour, Matthew L. Cartter

**Affiliations:** Yale University School of Public Health, New Haven, Connecticut, USA (J.L. Hadler);; Connecticut Department of Public Health, Hartford, Connecticut, USA (S. Petit, M. Mandour, M.L. Cartter)

**Keywords:** methicillin-resistant Staphylococcus aureus, MRSA, community-associated, hospital onset, health care–associated, invasive infection, trends, bacteria, antimicrobial resistance, Connecticut, United States, USA

## Abstract

Decreases in health care–related isolates accounted for all reductions in MRSA during 2007–2010.

## Medscape CME Activity

Medscape, LLC is pleased to provide online continuing medical education (CME) for this journal article, allowing clinicians the opportunity to earn CME credit.

This activity has been planned and implemented in accordance with the Essential Areas and policies of the Accreditation Council for Continuing Medical Education through the joint sponsorship of Medscape, LLC and Emerging Infectious Diseases. Medscape, LLC is accredited by the ACCME to provide continuing medical education for physicians.

Medscape, LLC designates this Journal-based CME activity for a maximum of 1 *AMA PRA Category 1 Credit(s)*^TM^. Physicians should claim only the credit commensurate with the extent of their participation in the activity.

All other clinicians completing this activity will be issued a certificate of participation. To participate in this journal CME activity: (1) review the learning objectives and author disclosures; (2) study the education content; (3) take the post-test with a 70% minimum passing score and complete the evaluation at www.medscape.org/journal/eid; (4) view/print certificate.

**Release date: May 15, 2012; Expiration date: May 15, 2013**

## Learning Objectives

Upon completion of this activity, participants will be able to:

- Identify the most common classification of MRSA recorded in 2001 to 2010
- Evaluate the epidemiology of MRSA between 2001 and 2010
- Distinguish the classification of MRSA which increased between 2001 and 2010
- Analyze the bacteriology of MRSA infections in the current study

## CME Editor

**Carol E. Snarey, MA,** Technical Writer/Editor, *Emerging Infectious Diseases. Disclosure: Carol E. Snarey, MA, has disclosed no relevant financial relationships.*

## CME Author

**Charles P. Vega, MD,** Health Sciences Clinical Professor; Residency Director, Department of Family Medicine, University of California, Irvine. *Disclosure: Charles P. Vega, MD, has disclosed no relevant financial relationships.*

## Authors

*Disclosures:* ***James L. Hadler, MD, MPH; Susan Petit, MPH; Mona Mandour, BS;*** *and* ***Matthew L. Cartter, MD, MPH,*** *have disclosed no relevant financial relationships.*
