# Supplementary material for: Trends in Invasive Infection with Methicillin-Resistant Staphylococcus aureus, Connecticut, USA, 2001–2010
Source: Emerg Infect Dis. 2012 Jun;18(6):917–24. doi: 10.3201/eid1806.120182 (PMC3358174; doi:10.3201/eid1806.120182)
Supplement: Related texts

# Research article: Trends in Invasive Infection with Methicillin-Resistant *Staphylococcus aureus,* Connecticut, USA, 2001–2010

**DOI:** 10.3201/eid1806.120182

**Authors:** James L. Hadler, Susan Petit, Mona Mandour, Matthew L. Cartter

**Affiliations:** Yale University School of Public Health, New Haven, Connecticut, USA (J.L. Hadler);; Connecticut Department of Public Health, Hartford, Connecticut, USA (S. Petit, M. Mandour, M.L. Cartter)

## Abstract

We examined trends in incidence of methicillin-resistant *Staphylococcus aureus* (MRSA) infections in Connecticut, with emphasis on 2007–2010, after legislation required reporting of hospital infections. A case was defined as isolation of MRSA from normally sterile body sites, classified after medical record review as hospital onset (HO), community onset, health care–associated community onset (HACO), or community-associated (CA). Blood isolates collected during 2005–2010 were typed and categorized as community- or health care–related strains. During 2001–2010, a total of 8,758 cases were reported (58% HACO, 31% HO, and 11% CA), and MRSA incidence decreased (p<0.05) for HACO and HO, but increased for CA. Significant 3- to 4-year period trends were decreases in all MRSA (–18.8%), HACO (–12.8%), HO (–33.2%), and CA (–12.7%) infections during 2007–2010, and an increase in CA infections during 2004–2006. Decreases in health care–related isolates accounted for all reductions. Hospital infections reporting may have catalyzed the decreases.

Methicillin-resistant *Staphylococcus aureus* (MRSA) was first identified in 1961 in England (*1*). The pathogen became a growing concern in US hospitals as *S. aureus* infections steadily increased, from causing 2.4% of nosocomial infections in 1975 to 29% by 1991 (*2*). In the mid-1990s, reports of community-onset infections caused by MRSA increased (*3**,**4*), including in children with no health care–associated risk factors (*5**,**6*). A 1998 population-based study in Connecticut showed that MRSA accounted for 23% of all blood isolates from persons with community-onset *S. aureus* infection admitted to the hospital (*7*). Subsequent studies showed that 2 epidemiologically distinct, although overlapping, MRSA problems were occurring, one in the hospital associated with highly antimicrobial drug–resistant MRSA strains and the other in the community. The infections in the community were fueled by 2 factors: persons’ exposure during health care to hospital-generated strains and the emergence and transmission of strains in the community that are less resistant to antimicrobial drugs (*8**,**9*).

In the late 1990s, the Emerging Infections Program (EIP), funded by the Centers for Disease Control and Prevention (CDC), became interested in better defining MRSA dynamics from a population-based perspective and established pilot surveillance systems at sites in 4 states for either all MRSA infections (Minnesota, Georgia, Maryland) or for invasive disease (Connecticut) (*8**,**10**,**11*). On the basis of findings from these efforts, formal population-based surveillance for invasive MRSA began in mid-2004 in 9 EIP sites comprising 16.5 million persons to obtain a national picture of the magnitude and trends in the most serious MRSA infections. A summary of data from 2005, the first full year of EIP invasive disease surveillance, was published in 2007 and revealed the full magnitude of invasive MRSA in the United States: ≈94,360 persons had invasive infections in 2005, and 18,650 patients died while hospitalized (*12*). This study also demonstrated that most MRSA infections (85%) were health care–associated, with 69% occurring in the community rather than in the hospital.

Since 2004, health care–associated infections have received increasing national attention. Efforts by patients’ advocate groups beginning in 2004 have resulted in the passage of legislation mandating that hospitals report infections to their state health department in 27 states, and in at least 12 states, legislation related to MRSA reporting, screening, or producing MRSA infection control plans (*13*). Since then, state-level involvement in health care–associated infections, including MRSA, has become commonplace. Given that hospital and community factors can affect trends in MRSA incidence, determining their net population level (as well as hospital-level) effects is crucial.

Connecticut began population-based surveillance for invasive MRSA infection in 2001, thus providing an opportunity to examine trends over a 10-year period. Our objectives in this analysis were twofold: 1) to describe the epidemiology of invasive MRSA in Connecticut and trends over time by place of illness onset (community vs. hospital) and relationship to health care, and 2) to describe MRSA strain subtypes associated with place of onset and trends over time.

## Methods

### Study Design

Connecticut participates in the Active Bacterial Core Surveillance project of the EIP at CDC. As part of this surveillance project, statewide active surveillance for invasive MRSA began in 2001. A case of invasive MRSA infection was defined as isolation of MRSA from a normally sterile body site (per Active Bacterial Core Surveillance protocol) (*10*) of a Connecticut resident at a clinical laboratory in Connecticut. Cases were identified through mandated statewide reporting by laboratories and clinicians. To verify completeness of reporting, we routinely audited all in-state hospital and reference laboratories. Cases for which a diagnosis was made out of state and those with cultures considered to be contaminants on the basis of a medical record discharge summary were excluded. Recurrent MRSA infections (positive culture taken >30 days after the initial positive culture in the same patient) were also excluded, both because the focus of this analysis was to ascertain the number and incidence of unique persons affected and because tracking for recurrence only began in 2004.

Medical chart reviews were conducted for hospitalized patients by using a standardized case report form. Data collected included demographics, infection type, underlying illnesses, and risk factors for infection as described below.

### Variable Definitions

Cases were classified into 3 mutually exclusive categories according to place of onset and relationship to health care. Hospital-onset (HO) MRSA cases were those for which cultures were collected >2 days after hospital admission (day of admission = day 0). Health care–associated community-onset (HACO) cases were those for which cultures were collected from outpatients or <2 days after hospital admission from patients with 1) a history of hospitalization (including admissions >24 hours’ duration), surgery, dialysis, or residence in a long-term care facility in the year before the culture date, or 2) a central venous catheter present at the time of culture. Patients with community-associated (CA) cases had none of the HO or HACO risk factors listed above.

Connecticut is divided into 169 towns. Large towns were defined as those with a population >100,000 (n = 5); medium-sized towns had a population of 50,000–99,999 as estimated in 2005 (n = 12); and small towns had a population <50,000 (n = 152). Connecticut has 32 acute care hospitals. Hospital volume was categorized according to total annual number of Connecticut resident bed-days in 2009 as reported to the Connecticut Office of Health Care Access. High-volume hospitals (n = 7) averaged >80,000 patient bed-days per year and included both Connecticut tertiary care hospitals; medium-volume hospitals (n = 12) averaged 40,000–79,999; and low volume hospitals (n = 13) averaged <40,000.

### Laboratory Methods

From April 1, 2005, through December 31, 2010, a sample of MRSA isolates from blood was systematically collected from 8 sentinel hospital laboratories (2 high-volume, 6 medium-volume hospitals). These laboratories represented 4 metropolitan areas. Isolates were only collected from specimens from case-patients who resided in towns in which >90% of residents received health care at 1 of the 8 hospitals. In 3 metropolitan areas, all available blood isolates were collected. In the fourth area, with both high-volume participating hospitals, collection was limited to 3 blood isolates per month from each laboratory.

Isolates were submitted to the Connecticut Department of Public Health Laboratory for confirmation of *S. aureus* by performing a catalase test using 3% hydrogen peroxide, followed by a tube coagulase test using rabbit plasma with EDTA (Becton Dickinson Microbiology Systems, Franklin Lakes, NJ, USA). If the tube coagulase result was equivocal, a rapid latex kit (Remel Products, Lenexa, KS, USA) was used to confirm the result. Kirby-Bauer disk diffusion with a cefoxitin disk was conducted to confirm methicillin resistance.

Isolates were subtyped by pulsed-field gel electrophoresis (PFGE) with the restriction endonuclease *Sma*I using the standard PulseNet method (*14*). PFGE patterns were electronically forwarded to CDC for analysis with BioNumerics version 4.01 (Applied Maths, Austin, TX, USA) and grouped into pulsed-field types by using Dice coefficients and 80% relatedness. Isolates with pulsed-field types USA300 (ST8-MRSA-4), USA400 (ST1-MRSA-4), USA1000 (ST59-MRSA-4), and USA1100 (ST30-MRSA-4) were considered community-related strains, per previous EIP protocol (*12*). Isolates with pulsed-field types USA100 (ST5-MRSA-2), USA200 (ST36-MRSA-2), and USA500–800 (ST8, ST45, ST72, and ST5-MRSA-4, respectively) were considered health care–related strains.

### Statistical Methods

Incidence rates by year were calculated by using US Census Bureau yearly population estimates, except for 2010, for which the 2010 census counts were used. Rates were calculated overall for each MRSA category and for strata defined by age and race/ethnicity within the categories. Rates for HO MRSA were also calculated by using hospital specific resident bed-days as reported to the Connecticut Office of Health Care Access for 2001–2009. For 2010, bed-days from 2009 were used because 2010 data were not available. One hospital was excluded from the bed-day analysis because that facility does not report patient bed-days to the Office of Health Care Access.

Analyses were performed using SAS version 9.2 (SAS Institute. Inc., Cary, NC, USA). Percentages of cases in different demographic categories were compared by using the χ^2^ test. Ten-year incidence trends were examined by using the χ^2^ test for linear trend. To examine changes in trends within the overall 10-year period, we analyzed trends by χ^2^ separately for 2001–2003, 2004–2006, and 2007–2010, in part because MRSA received substantial national publicity in 2007 after an article reported incidence at EIP sites (*12*), a high school student in Virginia died from MRSA (*15*), and many state legislatures passed laws mandating reporting of MRSA and/or hospital infections (*13*).

## Results

### Epidemiologic Findings

The incidence of MRSA overall and average annual incidence rates by each of the 3 categories are reported in Table 1. A total of 8,758 cases of MRSA infections were reported over the 10-year period, of which 920 (10.5%) were CA, 2,753 (31.4%) were HO, 5,075 (57.9%) were HACO, and 10 (0.1%) were undefined. The average annual incidence was 25.2 cases/100,000 population. Incidence sharply increased with age, with a >70-fold increase from 1.5/100,000 among children <18 years to 111.5/100,000 among adults >65 years (p<0.00001, χ^2^ for trend). The incidence in men was 1.6 times that in women. In addition, the incidence in non-Hispanic blacks was 1.4-fold higher than in whites, and the incidence in Hispanics was 0.64 that of whites. Furthermore, residents of large towns had 1.2 and 1.4 times the incidence of residents of medium-size and small towns.

**Table 1:** Cases and incidence rates of MRSA infection by place of onset and association with healthcare and demographic features, Connecticut, USA, 2001–2010*†

Demographic characteristic	All MRSA			HO			HACO			CA	
	No.	Rate		No.	Rate		No.	Rate		No.	Rate
Total	8,758	25.2		2,753	7.9		5075	14.6		920	2.6
Sex											
M	5,290	31.2		1,620	9.6		3,043	18.0		620	3.7
F	3,465	19.4		1,132	6.3		2,030	11.4		300	1.7
Age-group, y											
<18	127	1.5		65	0.8		25	0.3		37	0.4
18–34	379	5.3		115	1.6		148	2.1		116	1.6
35–49	1,082	13.0		308	3.8		529	6.4		244	2.9
50–64	1,955	31.4		642	10.3		1088	17.5		222	3.6
>65	5,205	111.5		1,619	34.7		3,279	70.2		301	6.4
Race/ethnicity‡											
White, non-Hispanic	4,649	25.6		1,338	7.4		2,765	15.2		537	3.0
Black, non-Hispanic	815	35.9		232	10.2		466	20.5		116	5.1
Hispanic	467	16.5		107	3.8		245	8.6		115	4.1
Town size§											
Large	1,930	31.6		597	9.8		1067	17.5		265	4.3
Medium	1,969	26.2		635	8.5		1,174	15.6		159	2.1
Small	4,841	22.9		1,511	7.1		2,826	13.3		496	2.3

*MRSA, methicillin-resistant *Staphylococcus aureus*; HO, hospital onset; HACO, health care–associated community onset; CA, community associated. †Incidence rates = average annual number of cases per 100,000 group-specific population using 2005 estimated population as the denominator. ‡Race-ethnicity only determined for all categories from 2004–2010. Denominator is 2007 estimated population. §Large >100,000 population; medium 50,000–99,999 population; small <50,000 population.

The descriptive epidemiology of HO, HACO, and CA MRSA infections had similarities and differences. For each, incidence was higher with increasing age, and in men, blacks, and large town residents (Table 1). However, persons with CA MRSA tended to be younger (43.2% <50 years vs. 13.8% and 17.7% for HACO and HO, p<0.00001 for each), and CA was the only 1 of these 3 groups in which incidence in Hispanics was higher than in whites (4.1/100,000 vs. 3.0/100,000, p = 0.01).

The overall incidence of MRSA infections by year and the yearly rates for each of the 3 categories of MRSA are shown in Figure 1. All have statistically significant trends from 2001–2010 (p<0.002): all MRSA (−14.2%), HO (−48.4%), and HACO (−3.8%) decreased while CA (+124.8%) increased. The trends were not constant over the entire 10 years, however. When 3-year intervals were considered (2001–2003, 2004–2006, and 2007–2010), the only statistically significant trends were decreases in all MRSA, HO, and HACO infections during 2007–2010 (all p<0.04) and an increase in CA during 2004–2006 (p = 0.001). During 2007–2010, overall MRSA incidence dropped 18.8%, HO dropped 33.2%, and HACO dropped 12.8%. During 2004–2006, CA increased 64.7%. However, during 2007– 2010, CA decreased 12.7% (p = 0.30).

**Figure 1:** Incidence of methicillin-resistant *Staphylococcus aureus* infection, by relationship to healthcare and year, Connecticut, USA, 2001–2010. CA, community onset; HACO, health care–associated community onset; HO, hospital onset.

When we assessed which demographic groups were most and least affected by these changes during 2007–2010, we found the following. For all MRSA infections during 2007–2010, a decrease >10% occurred in each demographic group. For HO MRSA, >10% decreases occurred in all groups except blacks (−7.3%). For HACO, >10% decreases occurred in all groups except those <18 years (+1%) and those 18–34 years of age (+14.2%). For CA, all groups showed decreases, except persons >50 years (+1%) and small town residents (+6.4%).

We also examined incidence of HO MRSA infections by hospital volume (Table 2). Increasing hospital volume was associated with higher average annual incidence (p<0.001). Overall, and for high- and medium-volume hospitals, incidence had a downward trend over the 10 years (p<0.0001 for each); the lowest rates were in 2009 and 2010 (Figure 2). No consistent 10-year trend was found for low-volume hospitals. When examined by the 3 periods (2001–2003, 2004–2006, 2007–2010), however, low-volume hospitals had increases in the first 2 periods (p = 0.04 and p = 0.07, respectively) and a decrease in the last (p = 0.01). Over time, rates based on hospital size have tended to converge, with a 3.9-fold difference in high-volume versus low-volume hospitals in 2001 reduced to 1.5-fold in 2010.

**Table 2:** Cases and incidence rates of hospital-onset MRSA infection by volume of hospital, at 29 acute care hospitals, Connecticut, USA, 2001–2010*

Hospital volume†	No. cases‡	Annual average incidence rate (range)§
Total	2,563	13.6 (8.9–18.1)
High	1,494	15.5 (10.1–23.3)
Medium	797	12.1 (6.7–17.1)
Low	272	10.1 (5.9–15.3)

*MRSA, methicillin-resistant *Staphylococcus aureus*. †High, >80,000 patient bed-days/year; medium, 40,000–79,999 patient bed-days/year; low, <40,000 patient bed-days/year. ‡Number of cases is <2,753 because data were excluded from 3 (N = 32) hospitals that either did not report MRSA and/or the number of patient bed-days for some years. §Annual average number of cases per 100,000 patient bed-days.

**Figure 2:** Incidence of hospital-onset methicillin-resistant *Staphylococcus aureus* infection, by hospital volume and year, Connecticut, USA, 2001–2010.

### Bacteriologic Findings

We examined the percentage of all MRSA isolates from the leading isolation sites by MRSA category. Blood isolates were most common (89.1%), followed by joint isolates (6.0%) and bone isolates (2.7%). CA infections were less likely than either HO or HACO infections to have a sterile-site isolate from blood (79.9% vs.88.2% and 91.2%, p<0.0001 for each), but more likely to have an isolate from a joint (18% vs. 2.0 and 5.9%, p<0.0001 for each). When 10-year trends were examined for each isolate site and place of onset category, 2 trends (at 2 sites) were significant. Joint isolates increased for HO and HACO MRSA infections, from 1.5% in 2001 to 5.4% in 2010 (p = 0.002) and from 4.7% in 2001 to 8.5% in 2010 (p = 0.005), respectively.

During 2005–2010, a total of 616 case-patients had blood isolates tested by PFGE at CDC; 609 had PFGE types in the range of USA100 to USA1100, inclusive. Of all PFGE types, 93 (15.3%) were community strains, of which USA300 was predominant (82, 88.2%). A total of 516 (84.7%) were health care strains, of which USA100 was predominant (456, 88.4%). There were 91 CA, 369 HACO, and 149 HO cases (Table 3). Isolates from CA cases were more likely than those from HACO and HO cases to have community PFGE types (49% vs. 11% and 6%, respectively), although the small difference in percentage between HACO and HO cases was statistically insignificant. During 2005–2010, isolates from CA and health care–associated (HACO and HO combined) cases were increasingly community PFGE types. The percentage of CA MRSA cases that were caused by community strain types increased from 28% to 56% (p = 0.02), and the percentage of health care–associated cases that were caused by community strains increased from 4% to 15% (p = 0.003).

**Table 3:** MRSA sterile site isolates, by PFGE type, place of onset category, and year, Connecticut, USA, 2005–2010*

MRSA category and PFGE type	2005	2006	2007	2008	2009	2010	Total	p value, trend
CA								
C strain	5 (28)	7 (47)	3 (30)	10 (63)	11 (69)	9 (56)	45 (49)	0.02
H strain	13 (72)	8 (53)	7 (70)	6 (38)	5 (31)	7 (44)	46 (51)	
HACO								
C strain	5 (6)	7 (9)	4 (7)	6 (11)	7 (12)	10 (21)	39 (11)	0.01
H strain	74 (94)	67 (91)	53 (93)	49 (89)	50 (88)	37 (79)	330 (89)	
HO								
C strain	0	0	2 (6)	3 (11)	4 (21)	0	9 (6)	0.07
H strain	45 (100)	5 (100)	30 (94)	26 (90)	15 (79)	19 (100)	140 (94)	
HA								
C strain	5 (4)	7 (9)	6 (7%)	9 (11)	11 (14)	10 (15)	48 (9)	0.003
H strain	119 (96)	72 (91)	83 (83)	75 (89)	65 (86)	56 (85)	470 (91)	

*Values are no. (%). MRSA, methicillin-resistant *Staphylococcus aureus*; PFGE, pulsed-field gel electrophoresis; CA, community-associated; HACO, health care–associated community onset; HO, hospital onset; HA, health care–associated (combined HACO and HO); C strain, community-related strain; H strain, health care–related strain.

To estimate trends in incidence of MRSA due to community and to health care strains, we applied the percentages of each to statewide incidence of CA and of health care–associated MRSA for each year during 2005–2010 (Table 4). For both CA and health care–associated MRSA, estimated incidence caused by community strains increased, while estimated incidence caused by health care strains decreased.

**Table 4:** Estimated incidence of MRSA, by category and strain PFGE type and year, Connecticut, USA, 2005–2010*†

MRSA category and PFGE type	2005	2006	2007	2008	2009	2010	Incidence difference, 2005–2010
CA							
Statewide incidence	3.25	3.36	3.53	3.54	3.47	3.08	−0.17
% C strain‡	27.8	46.7	30.0	62.5	68.8	56.3	
C strain incidence	0.90	1.57	1.06	2.21	2.39	1.73	+0.83
H strain incidence	2.35	1.79	2.47	1.33	1.08	1.35	−1.00
HA							
Statewide incidence	24.16	21.78	22.54	19.27	18.13	18.95	−5.21
% C strain	4.0	8.9	6.7	10.7	14.5	15.2	
C strain incidence	0.97	1.93	1.52	2.06	2.62	2.87	+1.90
H strain incidence	23.19	19.85	21.02	17.21	15.51	16.08	−7.11

*MRSA, methicillin-resistant *Staphylococcus aureus*; PFGE, pulsed-field gel electrophoresis; CA, community associated; C strain, community-related strain; H strain , health care–related strain; HA, health care–associated (combined hospital-associated community onset and hospital onset). †Incidence per 100,000 population. ‡Percentage based on isolate testing shown in Table 3.

## Discussion

Invasive MRSA infections comprise at least 2 distinct MRSA groups: those caused by strains that evolved and spread in hospitals over the past 4 decades, and those caused by strains that evolved in the community and ≈15 years ago began to spread widely, causing community and institutional outbreaks of skin and soft tissue infections (*8**,**9*). Our population-based study analyzed trends in invasive MRSA in Connecticut from the health care and community perspectives. The key findings include the following: 1) the downward trend, previously documented in EIP sites during 2005 to 2008 (*16*), continued through 2010 in incidence of health care–associated invasive MRSA infections of both community and hospital onset; 2) invasive MRSA infection incidence declined consistently in hospitals of all sizes, in the community, and in almost all demographic groups since 2007; 3) community-associated invasive MRSA may have peaked; and 4) community strains are making an increasing contribution to invasive MRSA in hospitals and in the community.

At least 6 recently published large-scale studies have examined incidence trends in MRSA in the United States during various periods in the past decade (*16**–**21*). Most examined community-associated or community-onset MRSA and found large increases in the number of MRSA infections (*17*) or hospital admissions (*18**–**20*) from 1999–2000 through 2005–2007. The only published large-scale studies to find a trend toward decreasing incidence are 2 CDC studies. A study of invasive MRSA incidence in 9 EIP sites (including Connecticut) comprising ≈15 million persons, found that during 2005–2008, HO and HACO incidence declined 9.4% and 5.7%%, respectively (*16*). A more focused study of central line–associated bloodstream infections in US intensive care units found a net decrease of 49.6% during 1997–2007, with all the decrease occurring since 2001 (*21*).

The data from Connecticut during the same periods are consistent with those of other trends studies: CA MRSA was generally increasing from 2001 to 2007 (+158%), and HO and HACO MRSA decreased during 2005–2008 (−20% each). The additional years of data in Connecticut help clarify the situation for hospital and health care–associated infections before and after the EIP study and the situation for CA infections since 2007. HO MRSA infections appeared to begin to decrease during 2003–2004 and have remained on a downward trajectory since, while HACO infections appeared to be increasing during 2001–2004 and then began a generally downward trend. CA MRSA, after the large and generally continuous increase during 2001–2007, plateaued during 2007–2009 and then dropped 12.2% in 2010. The net effect of these varying trends on overall invasive MRSA infection has been an irregular, but net increase in, incidence during 2001–2007, and a large, persistent drop since 2007.

The typing data suggest that these varying trends in Connecticut can be connected. The subset of blood isolates typed during 2005–2010 showed that the percentages of all HO, HACO, and CA infections caused by community PFGE types are increasing, and those caused by health care–associated types are decreasing. When the percentages of community and health care–associated strains were applied to the statewide incidence of CA and of health care–associated MRSA for those years, statewide community-strain estimated incidence increased over time in each category, while statewide health care–associated strain estimated incidence decreased in each. Thus, the decrease in invasive MRSA infection can likely be explained by a reduction in disease incidence with health care–associated strains, while disease and, presumably, prevalence of community strains continue to increase.

Although our surveillance data analysis cannot directly address why health care strains and related invasive disease decreased while community strains and related disease did not, several explanations are possible. Since 2006, Connecticut laws have been passed that required the reporting of hospital infections and MRSA control policies in hospitals (*22**,**23*). In addition, in 2007, the Journal of the American Medical Association published a highly publicized article describing the magnitude and public health importance of health care–associated MRSA (*12*), which included Connecticut data, and the death of a Virginia high school student (*15*) generated substantial local as well as national media coverage. Collectively, these events likely created a high level of awareness among hospitals regarding MRSA control and increased attention to preventive efforts such as screening, isolation, and handwashing. As a consequence, carriage of MRSA in patients discharged from the hospital, mostly health care–related strains, likely decreased, with subsequent reductions in health care–associated strain infections and in community infections resulting from community transmission of these strains.

In contrast, controlling community strains that occur outside the hospital is not as easy. Although proactive control efforts in institutions, including correctional facilities and sports facilities, should minimize the potential for institutional outbreaks, much community transmission occurs outside such settings. Thus, one could expect the sustained prevalence and continued transmission of community strains in the community with regular introduction in proportion to their incidence into health care settings and that their proportion of all MRSA infections would increase.

Although our data show a plateauing of CA MRSA, they also suggest that the increasing incidence of infection with community strains, mostly USA300, in Connecticut, is not over. The plateau effect appears to be because the rate of decrease in infections caused by health care strains is now equal to the rate of increase in community strains. Future trends will be determined in part by the relative dynamics of these MRSA strains.

Our study has several strengths: in particular, the surveillance is population-based, complete, enhanced by regular audits of all Connecticut laboratories for MRSA isolates, and of 10 years’ duration. In addition, strain typing was performed on a subset of blood isolates.

However, this study also has several limitations. First, some HACO cases may have been misclassified as CA because only the most recent medical record was reviewed, and patients were not interviewed. Second, our data could have been influenced by trends in obtaining blood cultures from patients with fever, although we do not know whether a trend exists toward obtaining fewer blood cultures on acutely ill, febrile patients, most of whom need hospitalization. Third, the sample of isolates characterized was not random and may not be representative of the state as a whole. However, it was a systematic sample obtained from 4 metropolitan areas that had a collective MRSA epidemiology mirroring that of the state as a whole. Fourth, the hypothesis that the sharp drop in health care–related MRSA incidence beginning in 2008 may be due to a combination of hospital and public awareness raised in 2006 and 2007 is based on ecologic information only. We have not systematically reviewed hospital screening, isolation, hand washing, and other relevant policies and practices to determine whether they changed before MRSA began to decrease in each hospital. Fifth, CA-MRSA strains mainly cause skin and soft tissue infections. Although overall invasive disease appears to be decreasing, the replacement of health care–associated MRSA strains with community strains could result in an increase in noninvasive infections. Finally, the Connecticut experience may not be generalizable to other states or geographic regions with potentially different MRSA dynamics.

In summary, after 7 years of stable to increasing incidence, HA, HO, and CA MRSA all have been decreasing since 2007, coincident with increased public, public health, and hospital attention. The decrease appears to be entirely due to a decrease in incidence of infections caused by health care–related strains of MRSA. Continued monitoring is needed to assess the sustainability of the apparent prevention gains.

## References

[1] Jevons MP. Celbenin-resistant staphylococci. BMJ. 1961;1:124–5. 10.1136/bmj.1.5219.124-a

[2] National Nosocomial Surveillance (NNIS) report, data summary from October 1986–April 1996, issued May 1996. Am J Infect Control. 1996;24:380–8. 10.1016/S0196-6553(96)90026-78902113

[3] Layton MC, Hierholtzer WJ, Patterson JE. The evolving epidemiology of methicillin-resistant *Staphylococcus aureus* at a university hospital. Infect Control Hosp Epidemiol. 1995;16:12–7. 10.1086/6469967897168

[4] Moreno F, Crisp HE, Patterson JE. Methicillin-resistant *Staphylococcus aureus* as a community organism. Clin Infect Dis. 1995;21:1308–12. 10.1093/clinids/21.5.13088589164

[5] Herold BC, Immergluck LC, Maranan MC, Lauderdale DS, Gaskin RE, Boyle-Vavra S, Community-acquired methicillin-resistant *Staphylococcus aureus* in children with no identified predisposing risk. JAMA. 1998;279:593–8. 10.1001/jama.279.8.5939486753

[6] Centers for Disease Control and Prevention. Four pediatric deaths from community-acquired methicillin-resistant *Staphylococcus aureus*: Minnesota and North Dakota, 1997–1999. MMWR Morb Mortal Wkly Rep. 1999;48:707–10.21033181

[7] Morin CA, Hadler JL. Population-based incidence and characteristics of community-onset *Staphylococcus aureus* infections with bacteremia in 4 metropolitan Connecticut areas, 1998. J Infect Dis. 2001;184:1029–34. 10.1086/32345911574918

[8] Naimi TS, LeDell KH, Como-Sabetti KM, Borchardt SM, Boxrud DJ, Etienne J, Comparison of community- and health care-associated methicillin-resistant *Staphylococcus aureus* infection. JAMA. 2003;290:2976–84. 10.1001/jama.290.22.297614665659

[9] Tenover FC, McDougal LK, Goering RV, Killgore G, Projan SJ, Patel JB, Characterization of a strain of community-associated methicillin-resistant *Staphylococcus aureus* widely disseminated in the United States. J Clin Microbiol. 2006;44:108–18. 10.1128/JCM.44.1.108-118.200616390957PMC1351972

[10] Fridkin SK, Hageman JC, Morrison M, Sanza LT, Como-Sabetti K, Jernigan JA, Methicillin-resistant *Staphylococcus aureus* disease in three communities. N Engl J Med. 2005;352:1436–44. 10.1056/NEJMoa04325215814879

[11] Petit S, Barrett NL, Fraser Z, Hadler JL. Epidemiology of community-onset invasive infection with methicillin-resistant *Staphylococcus aureus* (MRSA) in Connecticut, 2001–2004. In: International Conference on Emerging Infectious Diseases 2006: program and abstracts book. Washington (DC): American Society for Microbiology; 2006. p. 160.

[12] Klevens RM, Morrison MA, Nadle J, Petit S, Gershman K, Ray S, Invasive methicillin-resistant *Staphylococcus aureus* infections in the United States. JAMA. 2007;298:1763–71. 10.1001/jama.298.15.176317940231

[13] Committee to Reduce Infections Deaths. State legislation and initiatives on healthcare associated infections, updated October 2011 [cited 2011 Dec 19]. http://www.hospitalinfection.org/legislation.shtml.

[14] McDougal LK, Steward CD, Killgore GE, Chaitram JM, McAllister KS, Tenover FC. Pulsed-field gel electrophoresis typing of oxacillin-resistant *Staphylococcus aureus* isolates from the United States: establishing a national database. J Clin Microbiol. 2003;41:5113–20. 10.1128/JCM.41.11.5113-5120.200314605147PMC262524

[15] Davis R, Coughlin J. Governments urged to make killer bugs a priority. USA Today, October 19, 2007 [cited 2012 Apr 2]. http://www.usatoday.com/news/health/2007-10-18-super-bug-staph_N.htm

[16] Kallen AJ, Mu Y, Bulens S, Reingold A, Petit S, Gershman K, Health care-associated invasive MRSA infections, 2005–2008. JAMA. 2010;304:641–7. 10.1001/jama.2010.111520699455

[17] Como-Sabetti K, Harriman KH, Buck JM, Glennen A, Boxrud DJ, Lynfield R. Community-associated methicillin-resistant *Staphylococcus aureus*: trends in case and isolate characteristics from six years of prospective surveillance. Public Health Rep. 2009;124:427–35.1944541910.1177/003335490912400312PMC2663879

[18] Klein E, Smith DL, Laxminarayan R. Hospitalization and death rates caused by methicillin-resistant *Staphylococcus aureus*, United States, 1999–2005. Emerg Infect Dis. 2007;13:1840–6.1825803310.3201/eid1312.070629PMC2876761

[19] Gerber JS, Coffin SE, Smathers SA, Zaoutis TE. Trends in the incidence of methicillin-resistant *Staphylococcus aureus* infection in children’s hospitals in the United States. Clin Infect Dis. 2009;49:65–71. 10.1086/59934819463065PMC2897056

[20] Illinois Department of Public Health. MRSA trends in Illinois hospitals: 2002–2007 [cited 2012 Apr 2]. http://www.idph.state.il.us/health/infect/MRSA_Data_02-07.pdf

[21] Burton DC, Edwards JR, Horan TC, Jernigan JA, Fridkin SK. Methicillin-resistant *Staphylococcus aureus* central line–associated bloodstream infections in US intensive care units, 1997–2007. JAMA. 2009;301:727–36. 10.1001/jama.2009.15319224749

[22] State of Connecticut. Public act no. 06–142. An act concerning hospital acquired infections [cited 2012 Apr 2]. http://www.cga.ct.gov/2006/ACT/PA/2006PA-00142-R00SB-00160-PA.htm.

[23] State of Connecticut. Public act no. 08–12. An act concerning methicillin-resistant *Staphylococcus aureus* [cited 2012 Apr 2]. http://www.cga.ct.gov/2008/ACT/PA/2008PA-00012-R00SB-00579-PA.htm

# Research article

**DOI:** 10.3201/eid1806.120182

## Medscape CME Quiz

## Earning CME Credit

To obtain credit, you should first read the journal article. After reading the article, you should be able to answer the following, related, multiple-choice questions. To complete the questions (with a minimum 70% passing score) and earn continuing medical education (CME) credit, please go to www.medscape.org/journal/eid. Credit cannot be obtained for tests completed on paper, although you may use the worksheet below to keep a record of your answers. You must be a registered user on Medscape.org. If you are not registered on Medscape.org, please click on the New Users: Free Registration link on the left hand side of the website to register. Only one answer is correct for each question. Once you successfully answer all post-test questions you will be able to view and/or print your certificate. For questions regarding the content of this activity, contact the accredited provider, CME@medscape.net. For technical assistance, contact CME@webmd.net. American Medical Association’s Physician’s Recognition Award (AMA PRA) credits are accepted in the US as evidence of participation in CME activities. For further information on this award, please refer to http://www.ama-assn.org/ama/pub/category/2922.html. The AMA has determined that physicians not licensed in the US who participate in this CME activity are eligible for *AMA PRA Category 1 Credits*™. Through agreements that the AMA has made with agencies in some countries, AMA PRA credit may be acceptable as evidence of participation in CME activities. If you are not licensed in the US, please complete the questions online, print the certificate and present it to your national medical association for review.

## Article Title: Trends in Invasive Infection with Methicillin-Resistant *Staphylococcus aureus*, Connecticut, USA, 2001–2010

## CME Questions

**1. What was the most common classification of methicillin-resistant *Staphylococcus aureus* (MRSA) over the 10-year study period of the current research?**

A. Hospital onset (HO)

B. Health care-associated community onset (HACO)

C. Community-associated (CA)

D. Undefined

**2. MRSA was most common among which of the following patient groups?**

A. Younger patients

B. Non-Hispanic black patients

C. Hispanic patients

D. Residents of small towns

**3. Which classification of MRSA was the only one to increase between 2001 and 2010?**

A. All MRSA cases

B. HO

C. HACO

D. CA

**4. Which of the following statements regarding the bacteriology of MRSA infections in the present study is most accurate?**

A. Blood isolates of MRSA were the most common infection

B. Joint isolates were more likely in HACO vs CA cases of MRSA

C. Most PFGE types were community strains of MRSA

D. The prevalence of community pulsed-field gel electrophoresis (PFGE) types declined between 2005 and 2010

## Activity Evaluation

**Table:** 

1. The activity supported the learning objectives.				
Strongly Disagree				Strongly Agree
1	2	3	4	5
2. The material was organized clearly for learning to occur.				
Strongly Disagree				Strongly Agree
1	2	3	4	5
3. The content learned from this activity will impact my practice.				
Strongly Disagree				Strongly Agree
1	2	3	4	5
4. The activity was presented objectively and free of commercial bias.				
Strongly Disagree				Strongly Agree
1	2	3	4	5
